# Efficacy of the new β-D-glucan measurement kit for diagnosing invasive fungal infections, as compared with that of four conventional kits

**DOI:** 10.1371/journal.pone.0255172

**Published:** 2021-08-26

**Authors:** Yuuki Bamba, Kei Nagano, Hiroshi Moro, Hideyuki Ogata, Mariko Hakamata, Satoshi Shibata, Takeshi Koizumi, Nobumasa Aoki, Yasuyoshi Ohshima, Satoshi Watanabe, Takeshi Nakamura, Sugako Kobayashi, Yoshiki Hoshiyama, Toshiyuki Koya, Toshinori Takada, Toshiaki Kikuchi

**Affiliations:** 1 Department of Respiratory Medicine and Infectious Diseases, Niigata University Graduate School of Medical and Dental Sciences, Niigata, Japan; 2 Department of Clinical Laboratory, Niigata University Medical and Dental Hospital, Niigata, Japan; Wadsworth Center, UNITED STATES

## Abstract

**Background:**

Each of the currently available (1→3)-β-D-glucan (BDG) measurement kits follows a different measurement method and cut-off value. Comparisons of diagnostic performance for invasive fungal infections (IFIs) are desirable. Additionally, ecological considerations are becoming increasingly important in the development of new measurement kits.

**Methods:**

The plasma BDG levels in clinical samples were measured using the following currently available kits: the Fungitec G test MKII, the Fungitec G test ES, Fungitell, the β-Glucan test Wako, and the newly developed Wako kit (Wako-Eu). Wako-Eu uses a pre-treatment solution that conforms to European regulations for the registration, evaluation, authorisation, and restriction of chemicals. The values obtained for the samples using each kit were studied and compared.

**Results:**

Of the 165 patients evaluated, 12 had IFIs, including pneumocystis pneumonia, aspergillosis, and candidiasis. BDG values obtained using the kits were moderately correlated with each other. Clinical diagnoses of the evaluated cases indicated that 21 false positives were diagnosed by at least one kit. The sensitivity of the Fungitell kit was relatively low, but those of the other four were over 90%. The specificity was above 90% for all kits. For positive predictive value, the Wako and the Wako-Eu methods were superior to the others owing to fewer false positive results.

**Conclusions:**

The newly developed Wako-Eu method, which considers ecological concerns, shows diagnostic performance equivalent to that of its predecessor. To improve the diagnostic accuracy of IFIs, it is necessary to interpret the results carefully, giving due consideration to the characteristics of each measurement kit.

## Introduction

When treating IFIs, early diagnoses and prompt initiation of appropriate antifungal agents generally result in better outcomes [1]. However, a definitive diagnosis of an IFI is often difficult because most patients with IFIs tend to avoid invasive examinations due to the complicated nature of their clinical backgrounds. In addition, conventional culture methods have relatively low sensitivity and require a long turnaround time to yield positive results [2–4]. Due to these circumstances, non-culture diagnostics have assumed importance as diagnostic adjuncts for IFIs. One such diagnostic adjunct is the concentration of serum (1→3)-β-D-glucan (BDG), a component of the fungal cell wall, in the blood [5–8]. BDG measurement assays have been widely employed as useful serological diagnostic methods for IFIs, including aspergillosis [7,8], candidiasis [6,9], and *Pneumocystis jirovecii* pneumonia (PCP) [10]. Based on meta-analyses, the pooled sensitivity and specificity for diagnosing IFIs were 75–78% and 81–87%, respectively [11–13]. Although a BDG assay can be useful in clinical practice, substantial heterogeneity exists across different studies, and its limitations should be noted.

The BDG assay, an application of the Limulus amoebocyte lysate (LAL) cascade reaction, was first developed using a substance derived from the blood of the Japanese horseshoe crab, *Tachypleus tridentatus*, in 1995 [14]. Thereafter, several BDG measurement kits have been made available worldwide. Three other BDG measuring kits are currently available in Japan, including the Fungitec G test ES “Nissui”, the Fungitec G test MKII “Nissui”, and the β-Glucan test Wako. Until recently, the Fungitell β-D-glucan assay kit was the only BDG measurement kit available in Europe and the United States; however, the launch of the β-Glucan test Wako in Europe provided another option for BDG analyses. Additionally, the safety of measurement reagents for the human body and the environment has become an area of concern. Given this, Wako-Eu, a revised Wako kit intended for the European market that uses a pre-treatment solution that conforms to 2021 European regulations for registration, evaluation, authorisation and restriction of chemicals (REACH), is under development. With the advent of these new measurement methods, the need for comprehensive evaluation of their diagnostic performances has arisen.

Based on the above background, the primary objective of this study was to conduct a comprehensive comparison of the diagnostic performances of all currently available BDG measurement kits. Secondly, diagnostic performance of the new measurement kit, Wako-Eu, was evaluated.

## Materials and methods

### Ethics statement

This study was in compliance with the principles of the Helsinki Declaration as well as current ethical guidelines, and was approved by the ethics committee of Niigata University Medical and Dental Hospital (#2015–2431). The requirement for written informed consent was waived, because residual samples were used in the study. The purpose of the study and the opportunity to opt out were provided on the hospital’s website.

### Study design and settings

This cross-sectional study was conducted at the Niigata University Medical & Dental Hospital (Niigata, Japan) from August 2017 to March 2018. We collected residual plasma samples from patients who were hospitalized for clinically suspected IFIs and underwent plasma BDG concentration measurement. Clinical data collected from electronic medical records included age, gender, medical history, medications and isolated microorganisms.

### Diagnostic classification

According to the revised criteria of the European Organization for Research and Treatment of Cancer/Invasive Fungal Infections Cooperative Group and the National Institute of Allergy and Infectious Diseases Mycoses Study Group (EORTC/MSG), patients with candidiasis or aspergillosis were classified into 4 categories (proven, probable, possible, and non-IFI) regardless of their BDG values [15]. *P*. *jirovecii* was detected via microscopic examination (Wright-Giemsa staining, Toluidine Blue O, and/or Grocott-Gomori) and a polymerase chain reaction test (PCR) performed on bronchoalveolar lavage fluid (BAL) [16]. PCR tests were performed by BML Inc., (Kawagoe, Japan). A PCP diagnosis was made when (i) at least 2 of the 4 following factors were present: cough, fever (elevated above 38°C), dyspnoea or decrease in peripheral oxygen saturation, as well as radiological features consistent with PCP on a high-resolution computed tomography scan; (ii) a favourable outcome with Trimethoprim/sulfamethoxazole; and (iii) *P*. *jirovecii* confirmed by microscopic examination and PCR. Patients showing all 3 factors were categorized as proven PCP, and patients who exhibited only (i) and (ii) but not (iii) were defined as possible PCP. Proven, probable and possible IFI patients were defined as the IFI group and non-IFI patients were classified as the control group.

### BDG assay

Plasma BDG levels were measured using all 4 conventional kits, including the Fungitec G test ES “Nissui” (ES; Nissui Pharmaceutical Co., Ltd., Tokyo, Japan), the Fungitell β-D-glucan assay kit (FA; Associates of Cape Cod, MA, USA), the Fungitec G test MKII “Nissui” (MKII; Nissui Pharmaceutical Co.), the β-Glucan test Wako (Wako, Kinetic-turbidimetric technique; FUJIFILM Wako Pure Chemical Corporation, Osaka, Japan) and the newly developed Wako-Eu kit (Wako), according to each manufacturer’s instructions. The measurement principles of the five reagents rely on the same enzyme cascade extracted from Limulus (horseshoe crab) species. The ES, FA, and MKII tests are colorimetric, while the Wako and Wako-Eu tests are turbidimetric. Measurement of BDG using FA was entrusted to Mira Vista Diagnostics (Indiana, USA) and MK II to SRL Inc., (Tokyo, Japan). Measurements via ES, Wako, and Wako-Eu methods were conducted by the FUJIFILM Wako Pure Chemical Corporation (Osaka, Japan).

### Data analysis

Correlations between BDG levels estimated using the measurement kits were analysed via Spearman’s rank correlation test. Cohen’s kappa, sensitivity, specificity, positive predictive value (PPV), negative predictive value (NPV), and the likelihood ratio (LR) of each kit were calculated using cut-off values recommended by the manufacturers of these kits, which were 20, 80, 20, and 11 pg/mL for ES, FA, MKII, and Wako, respectively. The cut-off value for the conventional Wako kit, 11 pg/mL, was also used to evaluate the Wako-Eu kit. Furthermore, clinical backgrounds of the false-positive cases were investigated to ascertain whether they displayed any known risk factors for producing false-positives, including intake of human blood products (albumin, immunoglobulin, coagulation factors, plasma protein fractions), haemodialysis with cellulose-based membranes, usage of surgical gauze or other materials containing glucan, oesophageal candidiasis, intake of Chinese medicine and prescription drugs containing glucan [3,17–19]. Furthermore, we generated a receiver operating characteristic (ROC) curve for each kit and calculated the area under the curve (AUC). A value of p < 0.05 was considered statistically significant. All data were analysed using the JMP 13 (SAS Institute Inc., NC, USA).

## Results

### Patient characteristics

Of the 165 patients included in this study, 12 had IFIs as follows; 5 had PCP, 2 had candidemia, 1 had invasive pulmonary aspergillosis, 1 had aspergillus pseudomembranous tracheobronchitis, 1 had aspergillus empyema, 1 had candida peritonitis, and 1 had chronic pulmonary aspergillosis. Two PCP patients were diagnosed as possible IFIs. Patient characteristics are summarised (Table 1).

**Table 1:** Patient characteristics.

	n = 165
Age, median (IQR)	65(52–75)
Male, n (%)	85(51)
IFI, n (%)	12(7)
possible IFI, n	2
Under treatment for IFI, n	1
IFI	
PCP	5
Candidemia	2
Invasive pulmonary aspergillosis	1
Aspergillus pseudomembranous Tracheobronchitis	1
Aspergillus empyema	1
Candida peritonitis	1
Chronic pulmonary aspergillosis	1

IQR: Interquartile range, IFI: Invasive fungal infection, PCP: *Pneumocystis jirovecii* pneumonia.

### Comparison of BDG levels between the new detection kit and the four other kits

**Fig 1:**
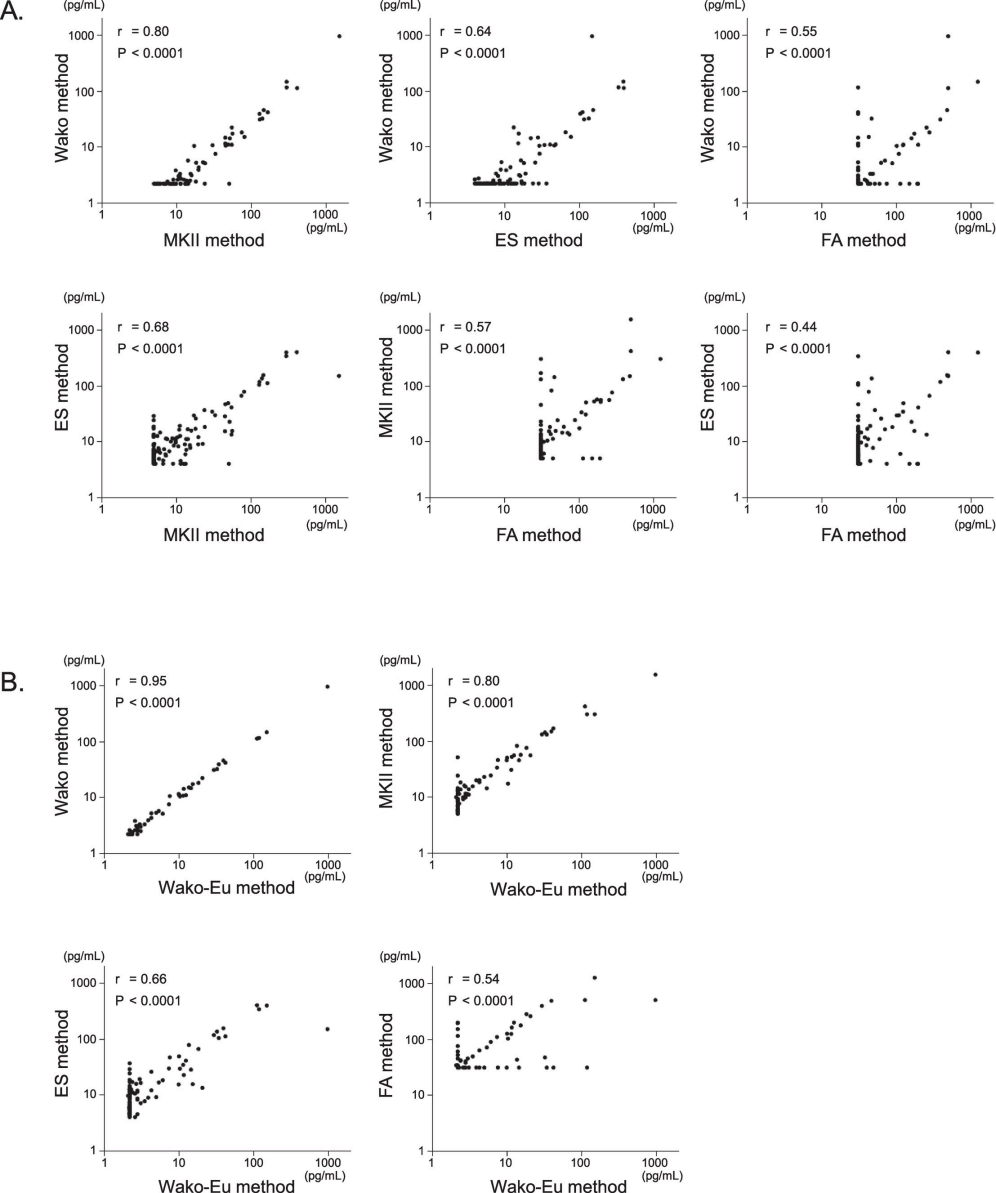
Comparison of BDG concentrations measured using each kit (N = 165).

We measured plasma BDG levels using the 4 conventional kits, ES, FA, MKII, and Wako, and the new kit (Wako-Eu). BDG values obtained from the conventional kits were moderately or strongly correlated. Correlations between FA and the other 3 kits were relatively low (Fig 1A). BDG levels measured using the Wako-Eu kit was most strongly correlated with the value measured using the conventional Wako kit, and was also either moderately or strongly correlated with the other conventional kits (Fig 1B). Concordance between these kits based on the manufacturer’s cut-off values is shown (S1 Table). Concordance between ES and FA, as well as between Wako and FA was lower than that between other combinations. In addition, Wako-Eu showed the highest match rate with Wako and modest match rates with the other 3 kits.

Measured BDG levels were compared between pre-existing kits (A) as well as between the new kit (Wako-Eu) and pre-existing kits (B). There was a very strong positive correlation between BDG values measured via the new Wako kit and those measured via the current Wako kit. The values obtained via the new kit were moderately to strongly correlated with the values obtained via other kits. Spearman’s rank correlation coefficient was used to examine the relationship between those kits.

BGD, β-D-glucan; Wako method, β-Glucan test Wako; Wako-Eu, European version of β-Glucan test Wako; MKII method, Fungitec G test MKII “Nissui”, ES method, Fungitec G test ES “Nissui”, and FA method, Fungitell β-D-glucan assay kit.

### Diagnostic efficacy

The measured values of each measurement method for the 12 IFI cases are shown in Table 2. On the other hand, clinical diagnoses indicated that 21 out of 165 patients were falsely determined to be positive by at least one kit (Table 3). Among them, only 8 had clinical risk factors (oesophageal candidiasis, post-surgery state, intake of traditional Chinese medicine and intake of intravenous immunoglobulin) that would lead to false positive determination via BDG measurement. Particularly, in the case of 7 false positive cases, only 1 out of 5 kits exceeded its cut-off values (016 for FP004, and 019 for ES, 010, 017, and 021 for FA, and 003 for MKII).

**Table 2:** Measured values of each measurement kit in IFIs.

	BDG (pg/mL)				
IFIs	ES	FA	MKII	Wako	Wako-Eu
PCP	154.4	484.0	148.0	45.2	39.7
PCP	337.4	31.0	300.0	115.2	119.2
PCP	134.8	47.0	142.0	31.9	32.6
PCP	116.7	392.0	131.0	30.8	29.6
PCP	397.1	500.0	415.0	112.1	112.1
Candidemia	77.6	43.0	81.8	15.0	13.7
Candidemia	13.3	257.0	55.3	22.1	20.8
Invasive pulmonary aspergillosis	22.5	161.0	52.1	14.2	11.7
*Aspergillus* pseudomembranous tracheobronchitis	149.2	500.0	1530.0	945.0	986.0
Aspergillus empyema	110.9	31.0	168.0	41.4	42.3
Candida peritonitis	103.7	31.0	130.0	38.9	34.4

Values below cut-offs are coloured in grey.

BGD, β-D-glucan; ES, Fungitec G test ES “Nissui” (cut-off >20 pg/mL); FA, Fungitell β-D-glucan assay kit (cut-off >80 pg/mL); MKII, Fungitec G test MKII “Nissui” (cut-off >20 pg/mL); Wako, β-Glucan test Wako (cut-off >11 pg/mL); Wako-Eu, European version of β-Glucan test Wako (cut-off >11 pg/mL).

**Table 3:** BDG values and possible causes of false positivity.

	BDG (pg/mL)								
FP Cases	ES	FA	MKII		Wako		Wako-Eu		Possible cause of FP
FP001	28.1	<31	45.2		14.6		14.6		Oesophageal candidiasis
FP002	15.3	<31	45.2		11.5		10.0		IVIG
FP003	9.1	<31	22.8		<6.0		<6.0		Unknown
FP004	25.8	63.0	18.4		<6.0		<6.0		Unknown
FP005	<4.0	197.0	51.0		<6.0		<6.0		Chinese medicine
FP006	15.5	176.0	56.5		17.2		15.4		Oesophageal candidiasis
FP007	392.4	1250.0	624.0		145.5		151.2		Post cardiac surgery
FP008	29.3	102.0	17.3		10.4		10.4		Post abdominal surgery
FP009	46.5	<31	45.7		10.5		7.6		Unknown
FP010	<4.0	193.0	<4.0		<6.0		<6.0		Unknown
FP011	34.3	124.0	30.5		10.7		11.5		Unknown
FP012	18.2	89.0	24.2		<6.0		6.1		Unknown
FP013	36.5	52.0	24.0		<6.0		<6.0		Unknown
FP014	29.6	109.0	33.4		7.5		7.4		Unknown
FP015	40.8	198.0	55.3		10.9		12.5		Unknown
FP016	28.7	<31	<4.0		<6.0		<6.0		Unknown
FP017	4.0	151.0	<4.0		<6.0		<6.0		Unknown
FP018	48.5	125.0	50.0		11.0		10.1		Post abdominal surgery
FP019	24.1	<31	<4.0		<6.0		<6.0		Unknown
FP020	65.9	280.0	75.2		18.1		18.4		Post abdominal surgery
FP021	6.0	114.0	<4.0		<6.0		<6.0		Unknown
Total number of FP cases	13	13	14		6		6		

Values above cut-offs are coloured in grey.

BGD, β-D-glucan; FP, false positive; IVIG, Intravenous Immunoglobulin; ES, Fungitec G test ES “Nissui”; FA, Fungitell β-D-glucan assay kit; MKII, Fungitec G test MKII “Nissui”; Wako, β-Glucan test Wako; Wako-Eu, European version of β-Glucan test Wako.

**Fig 2:**
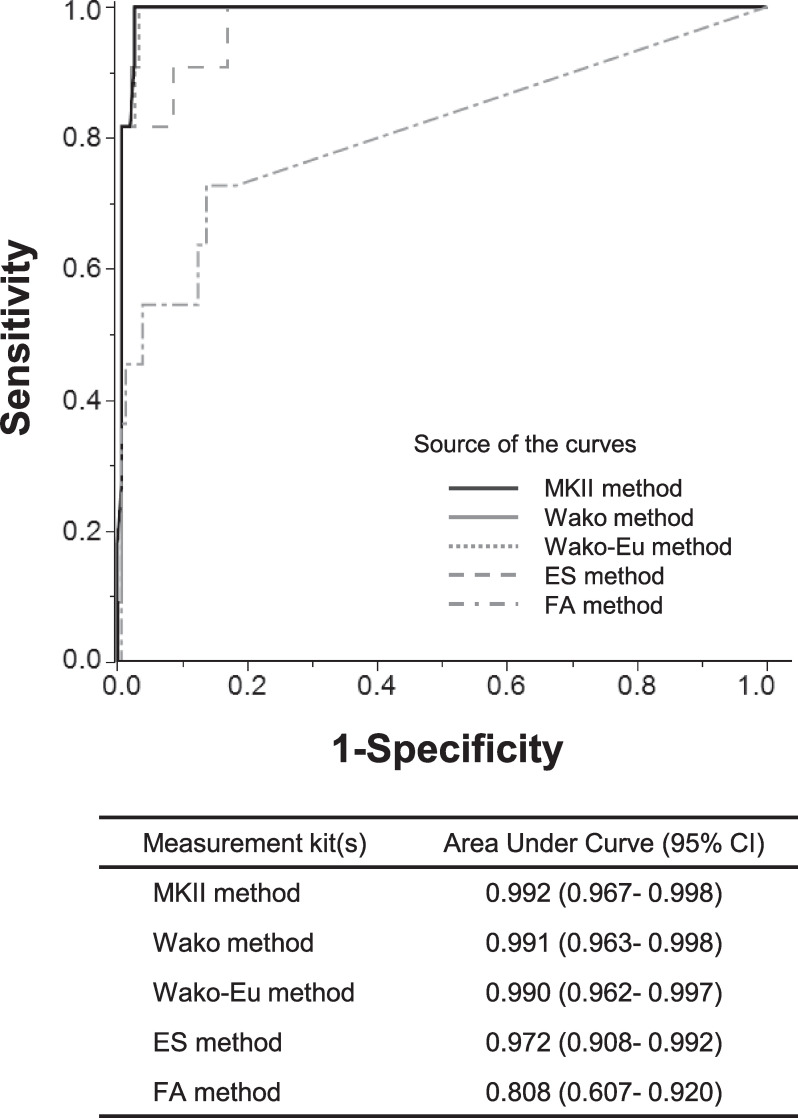
Receiver operating characteristic (ROC) curves of each BGD detection kit (N = 164).

Additionally, the sensitivity, specificity, PPV, NPV, positive LR, and negative LR of each kit for diagnosing IFIs, were calculated for 164 out of 165 cases; 1 case with chronic aspergillosis was not included (Table 4). The sensitivity of FA (58.3%) was very low, but those of the other 4 kits were above 90%. Specificity was above 90% in all kits. PPV, Wako and Wako-Eu were superior to the other methods with fewer false positives. For each kit, ROCs for diagnosing IFIs are shown (Fig 2). All BDG kits had high AUCs, but the AUC of FA was 0.816, a value which was inferior to those of other kits which were over 0.9.

**Table 4:** The diagnostic efficacy of each BDG detection kit (N = 164).

	Sensitivity (%)	Specificity (%)	PPV (%)	NPV (%)	LR+	LR-
ES	90.9 (10/11)	91.5 (140/153)	43.5 (10/23)	99.3 (140/141)	10.7	0.1
FA	63.6 (7/11)	91.5 (140/153)	35.0 (7/20)	97.2 (140/144)	7.5	0.4
MKII	100 (11/11)	90.8 (139/153)	44.0 (11/25)	100 (139/139)	10.9	0.0
Wako	100 (11/11)	96.1 (147/153)	64.7 (11/17)	100 (147/147)	25.5	0.0
Wako-Eu	100 (11/11)	96.1 (147/153)	64.7 (11/17)	100 (147/147)	25.5	0.0

BGD, β-D-glucan; PPV, Positive predictive value; NPV, Negative predictive value; LR+, Positive likelihood ratio; LR-, Negative likelihood ratio; ES: Fungitec G test ES “Nissui”; FA, Fungitell β-D-glucan assay kit; MKII, Fungitec G test MKII “Nissui”; Wako, β-Glucan test Wako; Wako-Eu, European version of β-Glucan test Wako.

ROC curves of BDG kits for the diagnosis of invasive fungal infection are shown. The area under curve of FA was inferior to those of other kits. One patient who was under treatment for chronic pulmonary aspergillosis was excluded.

BGD, β-D-glucan; Wako method, β-Glucan test Wako; MKII method, Fungitec G test MKII “Nissui”; ES method, Fungitec G test ES “Nissui”, and FA method, Fungitell β-D-glucan assay kit.

## Discussion

The current study measured plasma β-D-glucan levels of clinical samples using 5 commercially available kits, which were MKII, ES, FA, Wako, and the newly developed Wako-Eu. Although the measured BGD values did not exhibit a one-to-one correspondence between each pair of kits, a medium to strong correlation was noted between the kits. Notably, the Wako-Eu method showed a strong correlation with the conventional Wako method. As for diagnostic performance, the ES and MKII methods showed superior sensitivities of over 90%, whereas the Wako and Wako-Eu methods exhibited relatively higher specificities and positive predictive values. In contrast, the ES, FA, and MKII methods had more false positives, resulting in lower positive predictive values. Our results closely resembled those of previous studies [20–24]. According to previous studies conducted in Japan, MKII (or its predecessor, Fungitec G test MK) and ES exhibited superior sensitivity, while Wako showed superior specificity [20–22]. FA is the only BDG measurement kit commercially available in the United States and Europe. Recently, Wako has been made available in European countries, and a recent study comparing diagnostic performances for PCP has shown that, while FA was superior in sensitivity, Wako was superior in specificity [23].

All BDG measurement kits are based on LAL cascade reactions, although each uses a different pre-treatment method, standard substance, and quantification method, leading to specific cut-off values. Blood samples may contain endotoxins or other inhibitors or enhancers of Limulus reactions, and, therefore, each measurement kit utilizes pre-treatments to deactivate these substances. For this process, ES, FA, and MK II use alkaline solutions, while Wako/Wako-Eu methods use heat and non-ionic detergents with polymyxin B. Reportedly, reactivity to BDG varies depending on the species of horseshoe crabs [25]; *Limulus polyphemus* in North America or *T*. *tridentatus* in East Asia. However, horseshoe crab populations are in decline [26], making it difficult to obtain blood from *T*. *tridentatus*. Thus all 5 kits included in this study used substances derived from *L*. *polyphemus*. To quantify blood BDG levels, colorimetry is used in ES, FA, and MKII, while turbidimetry is used in Wako/Wako-Eu [27]. Measured values were fundamentally different between the two methods and, in the current study, the turbidimetry tended to yield lower values. Prior comparisons revealed that the colorimetric method was superior in sensitivity, while the turbidimetric method has fewer nonspecific reactions, leading to relatively higher specificity and a positive predictive value [21]. False positives associated with the BDG test are often problematic, and various factors have been reported to cause false positives [17–19]. Contamination of BGD, via blood products or post-surgery factors, affects all measurement kits, but contaminated samples accounted for only 9.5% of all false positives in our study. Therefore, unknown method-specific factors that cause nonspecific reactions may exist. Basically, although high sensitivity is crucial for early diagnosis of IFIs, frequent false positives may lead to the overuse of antifungal agents. Therefore, a proper understanding of all characteristics of the measurement kits is crucial so that BDG measurement results may be precisely interpreted, leading to the appropriate use of antifungal agents.

Certain limitations were associated with the present study. Firstly, it was conducted at a single centre, the university hospital. Thus, the possibility of unintentional selection bias could not be ruled out. Secondly, the sample of IFI patients used in the study was small. Furthermore, the majority of IFI patients had PCP, which tends to be associated with high BDG. Such patient configurations may affect the evaluation of measurement results. Thirdly, although FA instructions recommend the use of serum samples, plasma samples were used in all kits, and this may have affected the analysis. In contrast to European BGD assays, plasma samples, obtained via endotoxin-free sample tubes, are generally used for BGD assays in Japan. Except for FA, the measurement kits are designed for the analysis of plasma samples. Prior reports have compared FA and the other measurement kits using plasma samples as well as serum samples [21,23], and the differences between the two materials do not appear to affect the performance of the kits [23,24].

In conclusion, we compared the diagnostic performances of 5 BDG assay kits. The results of BDG tests should be interpreted relative to the characteristics of each kit. Ecological considerations have become increasingly important, and an improved Wako kit which conforms to REACH regulations is currently under development. This kit has been proven to be a promising alternative to its predecessor kit. However, further evaluation with more clinical samples as well as international standardization of BDG measurement methods are needed.

## Supporting information

S1 Table The concordance rate (*κ*) of each BDG test (N = 165). (DOCX)

S1 Dataset (XLSX)
