# Supplementary material for: Efficacy of the new β-D-glucan measurement kit for diagnosing invasive fungal infections, as compared with that of four conventional kits
Source: PLoS One. 2021 Aug 26;16(8):e0255172. doi: 10.1371/journal.pone.0255172 (PMC8389481; doi:10.1371/journal.pone.0255172)
Supplement: S1 Table — (DOCX) [file pone.0255172.s001.docx]

## Table S1. The concordance rate (𝜅) of each BDG test (N = 165)

|  |  | MKII |  |  |
| --- | --- | --- | --- | --- |
|  |  | Negative | Positive | Total |
| Wako | Negative | 140 | 8 | 148 |
|  | Positive | 0 | 17 | 17 |
| Total |  | 140 | 25 | 165 |
| 𝜅= 0.78 |  |  |  |  |
|  |  |  |  |  |
|  |  | FA |  |  |
|  |  | Negative | Positive | Total |
| Wako | Negative | 139 | 9 | 148 |
|  | Positive | 6 | 11 | 17 |
| Total |  | 145 | 20 | 165 |
| 𝜅= 0.54 |  |  |  |  |
|  |  |  |  |  |
|  |  | ES |  |  |
|  |  | Negative | Positive | Total |
| Wako | Negative | 139 | 9 | 138 |
|  | Positive | 3 | 14 | 17 |
| Total |  | 142 | 23 | 165 |
| 𝜅= 0.65 |  |  |  |  |
|  |  |  |  |  |
|  |  | ES |  |  |
|  |  | Negative | Positive | Total |
| MKII | Negative | 136 | 4 | 140 |
|  | Positive | 6 | 19 | 25 |
| Total |  | 132 | 23 | 165 |
| 𝜅= 0.75 |  |  |  |  |
|  |  |  |  |  |
|  |  | FA |  |  |
|  |  | Negative | Positive | Total |
| ES | Negative | 135 | 7 | 142 |
|  | Positive | 10 | 13 | 23 |
| Total |  | 145 | 20 | 165 |
| 𝜅= 0.54 |  |  |  |  |
|  |  |  |  |  |
|  |  | FA |  |  |
|  |  | Negative | Positive | Total |
| MKII | Negative | 136 | 4 | 140 |
|  | Positive | 9 | 16 | 25 |
| Total |  | 145 | 20 | 165 |
| 𝜅= 0.66 |  |  |  |  |
|  |  |  |  |  |
|  |  | Wako-Eu |  |  |
|  |  | Negative | Positive | Total |
| Wako | Negative | 146 | 2 | 148 |
|  | Positive | 2 | 15 | 17 |
| Total |  | 148 | 17 | 165 |
| 𝜅= 0.87 |  |  |  |  |
|  |  |  |  |  |
|  |  | Wako-Eu |  |  |
|  |  | Negative | Positive | Total |
| ES | Negative | 140 | 2 | 142 |
|  | Positive | 8 | 15 | 23 |
| Total |  | 148 | 17 | 165 |
| 𝜅= 0.72 |  |  |  |  |
|  |  |  |  |  |
|  |  | Wako-Eu |  |  |
|  |  | Negative | Positive | Total |
| MKII | Negative | 140 | 0 | 140 |
|  | Positive | 8 | 17 | 25 |
| Total |  | 148 | 17 | 165 |
| 𝜅= 0.78 |  |  |  |  |
|  |  |  |  |  |
|  |  | Wako-Eu |  |  |
|  |  | Negative | Positive | Total |
| FA | Negative | 140 | 5 | 145 |
|  | Positive | 8 | 12 | 20 |
| Total |  | 148 | 17 | 165 |
| 𝜅= 0.60 |  |  |  |  |

BGD, β-D-glucan; Wako, β-Glucan test Wako (cut-off >11 pg/mL); MKII, Fungitec G test MKII “Nissui” (cut-off >20 pg/mL); ES, Fungitec G test ES “Nissui” (cut-off >20 pg/mL); FA, Fungitell β-D-glucan assay kit (cut-off >80 pg/mL); Wako-Eu, European version of β-Glucan test Wako (cut-off >11 pg/mL).
